# Extensive Unprovoked Thromboembolism in Steroid-Dependent Ulcerative Colitis: A Case Report

**DOI:** 10.7759/cureus.38148

**Published:** 2023-04-26

**Authors:** Hassaan Arshad, Mohammad Abu-Abaa, Sindhu Chadalawada, Salman Kananeh

**Affiliations:** 1 Internal Medicine, Capital Health Regional Medical Center, Trenton, USA

**Keywords:** acute kidney injury, dvt, thrombosis, ulcerative colitis (uc), inflammatory bowel disease

## Abstract

The elevated risk of thromboembolism (TE) in association with inflammatory bowel disease (IBD) is well-established in literature. Herein, we present a case of a 70-year-old patient with steroid-dependent ulcerative colitis who presented with exertional dyspnea and abdominal pain. Investigations revealed extensive bilateral iliac and renal and caval venous thrombosis as well as pulmonary emboli. In addition to the rarity of such a finding in this location, this case serves to remind clinicians of the elevated risk of TE in those with IBD, even among those with IBD that has been in remission, especially in those presenting with unexplained abdominal pain and/or renal injury. TE can be life-threatening and requires a high index of clinical suspicion to establish early diagnosis and prevent propagation.

## Introduction

Inflammatory bowel disease (IBD) is a systemic disease characterized by inflammation of the gastrointestinal tract as well as involvement in extra-intestinal systems. It includes two major entities, ulcerative colitis and Crohn's disease. Thrombosis is a rare extra-intestinal manifestation of IBD, but it is well documented in the literature [1,2]. Patients with IBD have a more than three-fold increase in the risk of deep vein thrombosis (DVT) and pulmonary embolism (PE), which further increases to eightfold in case of flare even after correction for other prothrombotic factors [1,2]. Patients with IBD who are maintained on long-term steroids have an even higher risk of TE [3].

## Case presentation

**Figure 1:**
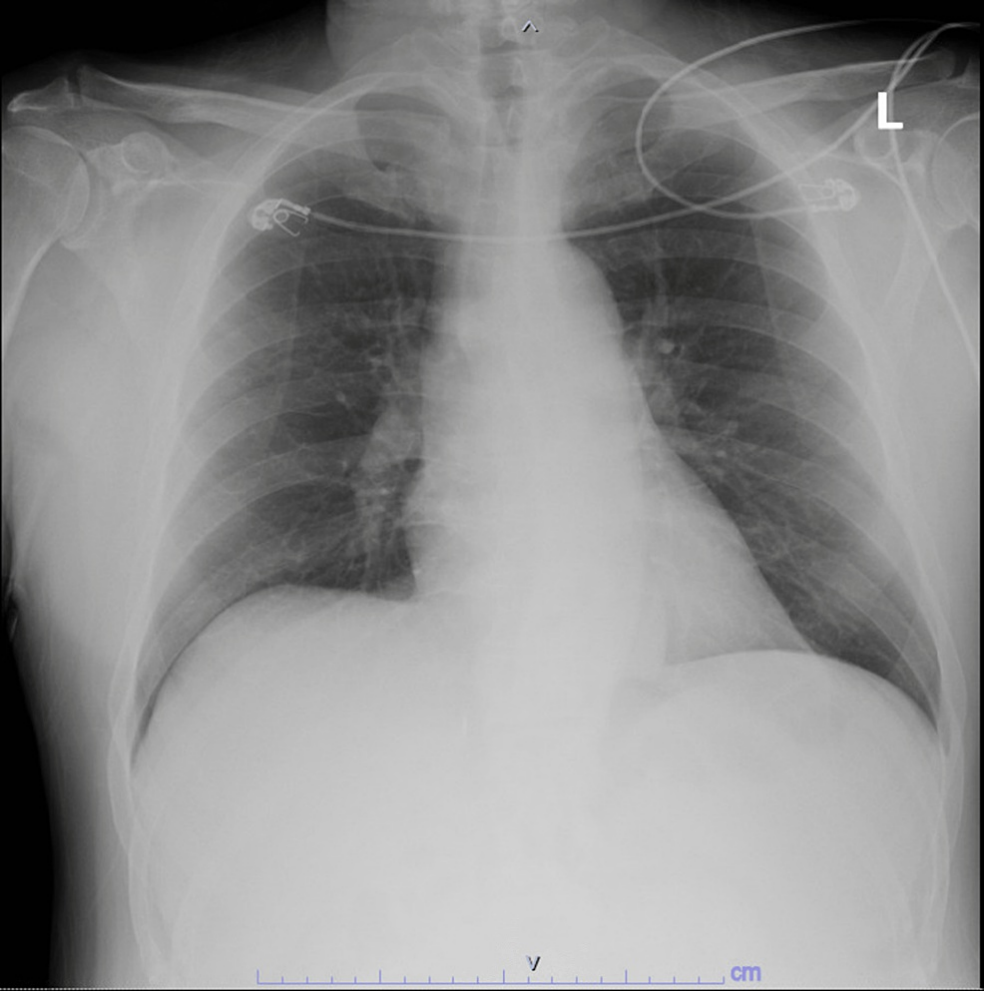
Chest X-ray

**Figure 2:**
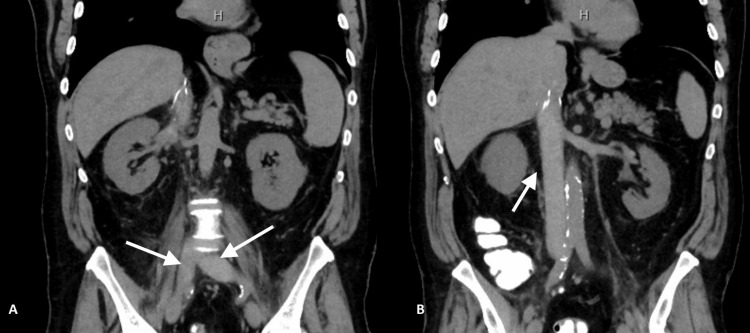
CT scan of the abdomen and pelvis CT scan coronal section showing evidence of fullness of bilateral common iliac veins (arrows in A) and the inferior vena cava (arrow in B) suggestive of extensive thrombosis.

**Figure 3:**
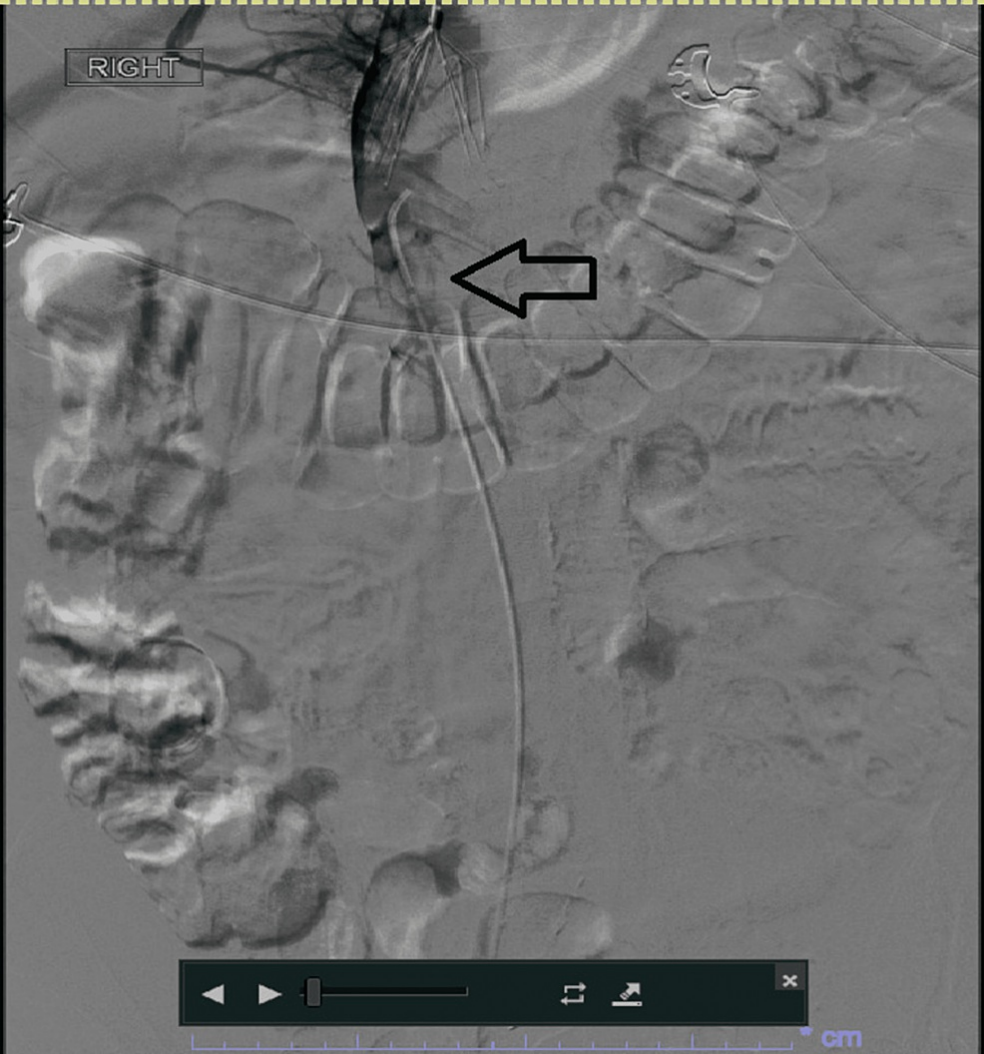
Initial venogram Initial venogram showed no flow of the contrast down the inferior vena cava below the injection site, confirming extensive inferior vena cava thrombosis (arrow).

A 70-year-old male patient presented to the emergency department (ED) with exertional shortness of breath and abdominal pain that was described as severe, cramping, and intermittent left lower quadrant pain. He denied any recent change in bowel habits, fever, or any evidence of bleeding. Past medical history was significant for pancolonic ulcerative colitis in clinical remission and on oral budesonide 4 mg daily with no extra-intestinal manifestations history and distant history of unprovoked pulmonary embolism and deep venous thrombosis during a flare of ulcerative colitis with inferior vena cava (IVC) filter placement during to gastrointestinal bleeding ten years prior to the current presentation. In ED, vital signs included a temperature of 37 degrees Celsius, heart rate of 82 beats per minute, respiratory rate of 17 cycles per minute, blood pressure of 120/70 mmHg, and SpO2 of 96% on room air. On the physical exam, the abdomen was soft and non-distended with no localized tenderness. Otherwise, the physical exam was unremarkable. Labs showed evidence of acute kidney injury (AKI) with elevated creatinine of 3.18 mg/dl with a normal baseline creatinine and elevated blood urea nitrogen (BUN) of 47 mg/dl. Otherwise, the labs were non-significant. The chest X-ray was unremarkable (Figure 1). Computed tomography (CT) scan of the abdomen and pelvis without contrast showed evidence of extensive thrombosis extending from suprarenal IVC to bilateral iliac veins and bilateral renal veins with no evidence of active colitis (Figure 2). Perfusion study of the lungs was pursued and showed evidence of subsegmental right-sided pulmonary embolism (Figure 3).

**Figure 4:**
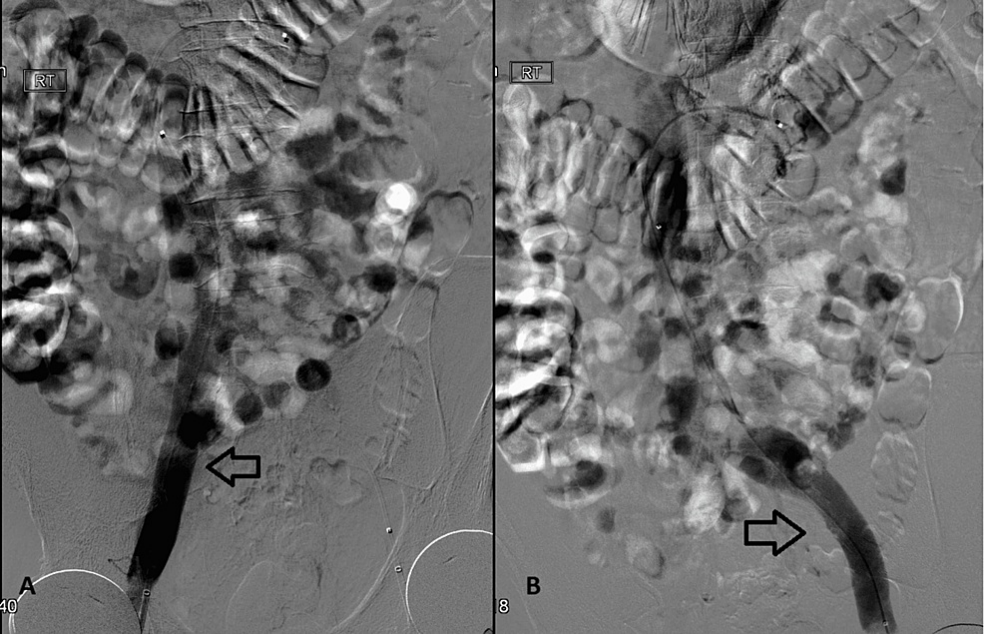
Post-thrombolysis venogram A follow-up venogram showing a clear flow of the contrast after effective thrombolysis (arrows in A and B).

The patient had a venogram with successful thrombolysis of the IVC, iliac and renal veins and he was started on heparin infusion with a 4000 units bonus and 1000 units/hour infusion rate. Gradual improvement in clinical features as well as renal function was achieved after the clearance of clot burden. Hypercoagulability screening, including anti-cardiolipin IgA, IgG, and IgM, factor V Leiden mutation, prothrombin mutation, homocysteine, protein C resistance, protein S and C function and level, Lupus anticoagulant and antithrombin was negative. He was started on apixaban 5 mg twice daily. Stability of clinical status and lack of evidence of bleeding on anticoagulation allowed for safe discharge home. A follow-up venogram one month later showed stability of venous vasculature (Figures 3, 4).

## Discussion

The first report of thromboembolism in inflammatory bowel disease (IBD) was in 1936 [4]. Thromboembolism (TE) has been well documented in the literature in association with IBD in multiple locations, including cerebral, retinal, pulmonary, cardiac, portal, and mesenteric arterial and venous vasculature [5]. The incidence rate of TE in Crohn's disease is elevated at 40/10,000 and 50/10,000 in ulcerative colitis, as compared to 10/10,000 in the general population, and the age of presentation tends to be younger among those with IBD as well [1]. There is also a gender difference, with the risk of TE higher in males compared to females [6]. Generally, the occurrence of TE is observed in active IBD, although one-third of cases happen during remission in well-controlled IBD [6]. 

Pathogenesis of TE in IBD is unclear [6] but likely involves interaction between genetic and environmental factors. Environmental factors like dehydration, long-term steroids, and immobilization may play a role, but the prevalence of genetic factors like factor V Leiden, prothrombin mutations, and protein C resistance is not increased among those with IBD [7]. As a systemic inflammatory disease, IBD is associated with elevated factor 8 activity, fibrinogen level, and thrombin production. There is also evidence of enhanced platelet activation and spontaneous aggregation with elevated thromboxane B2 and beta thromboglobulin levels [8,9]. It remains debatable if this coagulation cascade activation is a result of inflammatory status or an intrinsic feature of IBD [6]. Interestingly, our patient had no evidence of active disease and has been in remission for years prior to presentation. This supports the previous suggestion of IBD as an independent risk factor for TE [6]. 

Long-term steroid use causes a hypercoagulable state by the induction of increased procoagulant factors with coagulation cascade activation and impaired fibrinolytic capacity, which causes shortened partial thromboplastin time and increased time to clot lysis [10]. It is estimated that the risk of TE is elevated in long-term users of steroids by two to threefold [3,11]. 

Although TE is well established in association with IBD, the location (combined inferior vena cava, bilateral renal and iliac veins) and extent of thrombosis in our patient is quite rare and has been occasionally reported in the literature [12]. In this location, thrombosis of inferior vena cava (IVC), and renal and iliac veins can be subtle and vague and thus can be easily overlooked. This case serves to remind clinicians to remain vigilant of this possibility if a patient presents with unexplained abdominal pain and/or renal injury. 

The current recommendations for the treatment of TE in IBD are similar to other patients and involve weighing the risk of bleeding against the risk of TE. However, the duration remains unclear. The Canadian Association of Gastroenterology perceives the proinflammatory state of active IBD flare as a trigger for TE and thus recommends a duration of three months after flare resolution [13]. No current recommendation is available for primary prophylaxis of TE in those with IBD. However, in our patient, the need for lifelong anticoagulation was essential, given the extent and recurrent nature of TE.

## Conclusions

IBD is associated with an elevated risk of TE, even in remission. Long-term steroid use is associated with even higher risk. Although it is a rare site of thrombosis, unexplained abdominal pain and/or renal injury in those with a history of IBD should raise the consideration of IVC and/or renal vein thrombosis. Clinicians should maintain a high index of suspicion, allowing for earlier diagnosis and helping prevent propagation by early initiation of thromboprophylaxis if so deemed.
